# OCT Angiography of the Retina and the Choroid in the Macula in Patients with Normal Tension Glaucoma and Primary Open Angle Glaucoma

**DOI:** 10.3390/diagnostics14141485

**Published:** 2024-07-11

**Authors:** Anna Dastiridou, Maria Samouilidou, Eleftherios Anastasopoulos, Spyridon Koronis, Paraskevi Riga, Andreas Katsanos, Nikolaos Ziakas, Sofia Androudi

**Affiliations:** 1 Ophthalmology Clinic, University Hospital of Larissa, University of Thessaly, 41110 Larissa, Greece; androudi@otenet.gr; 2 2nd Ophthalmology Clinic, Aristotle University of Thessaloniki, 56403 Thessaloniki, Greece; msamouilidou@hotmail.com (M.S.); eleftherios70@gmail.com (E.A.); spyridonkoronis@gmail.com (S.K.); vivi.riga.p@gmail.com (P.R.); nikolasziakas@gmail.com (N.Z.); 3 Ophthalmology Clinic, University Hospital of Ioannina, University of Ioannina, 45500 Ioannina, Greece; katsanos@uoi.gr

**Keywords:** OCT angiography, open angle glaucoma, normal tension glaucoma, vessel density, superficial vascular plexus, choriocapillaris, choroid, blood flow

## Abstract

The aim of this study was to compare vessel density (VD) in the retina and choroid in eyes with primary open angle glaucoma (POAG), normal tension glaucoma (NTG) and controls. Patients with POAG, NTG and controls underwent OCT scanning of the macula and the disc followed by 6 × 6 mm macula OCT angiography (OCTA) imaging. Global and hemifield VD were recorded for the superficial (SVP) and deep (DVP) vascular plexus and the choriocapillaris (CC). The OCT thickness of the nerve fiber layer (NFL) and ganglion cell layer (GCC) was also measured. Data from 65 POAG, 33 NTG and 40 control eyes matched for age were analyzed. Mean SVP VD was lower in NTG and POAG eyes compared to controls (38.8 ± 5.3, 40.7 ± 6.8 and 48.5 ± 4.0%, *p* < 0.001). Mean DVP VD was lower in NTG and POAG eyes compared to controls (43.1 ± 6.1, 44.5 ± 7.6 and 48.6 ± 5.8%, *p* = 0.002). There was no difference in SVP VD or DVP VD between the glaucoma groups (*p* > 0.050). No difference was noted in CC VD between the groups (68.3 ± 2.3, 67.6 ± 3.7 and 68.5 ± 2.6%, *p* = 0.287). Lower SVP and DVP VD was seen in eyes with glaucoma compared to normal eyes. NTG and POAG eyes had similar VD loss. Eyes with glaucoma manifested similar CC VD compared to controls.

## 1. Introduction

Glaucoma is a multifactorial optic neuropathy that remains a major cause of irreversible vision loss [1]. Elevated intraocular pressure (IOP) is the most prominent risk factor for glaucoma and constitutes the primary therapeutic target for drugs, laser treatment or surgery. Nonetheless, glaucoma may progress despite an IOP within the statistically normal range, as encountered in primary open angle glaucoma (POAG) cases despite treatment and in normal tension glaucoma (NTG), a glaucoma subtype where untreated IOP does not exceed the level of 21 mmHg. The latter is especially common in Asia and less so in Caucasian populations [2]. It is now understood that factors independent of IOP contribute to the pathogenesis of glaucoma, at least in some patients. A vascular component has been suggested, with supporting evidence showing vascular dysregulation and reduced retrobulbar blood flow, especially in the normal tension glaucoma phenotype. Such studies linked glaucoma with decreased nailfold capillary blood flow, Raynaud’s syndrome and migraine, among other factors [3].

This association of glaucoma with vascular factors has also sparked an interest in investigating their prominence in NTG compared to high tension POAG. The earliest studies demonstrated the correlation of glaucomatous damage with decreased optic nerve head (ONH) perfusion utilizing methods such as laser Doppler flowmetry, color Doppler imaging and fluorescein angiography, but no significant differences between glaucoma subtypes were reported [4,5,6].

The advent of optical coherence tomography angiography (OCTA) has offered a new, non-invasive imaging technique that allows the evaluation and quantification of retinal and also choroidal vasculature. Through successive OCT scans at the same anatomic location, OCTA can detect the signal of the blood flow and image the retinal superficial and deep vascular plexus (SVP and DVP) of the macula, as well as the choriocapillaris (CC) [7]. OCTA can also characterize the vasculature in the optic nerve head and quantify the peripapillary retinal perfused capillaries (pRPCs), which constitute the innermost layer of capillaries, and which are located in the retinal nerve fiber layer (NFL). This technique surpasses the resolution capabilities of previously available modalities, which were limited to imaging only the larger retinal vessels. The newly developed algorithms have rendered the quantification of macular and ONH vessel density (VD) possible. Indeed, projection resolved OCTA has provided the opportunity to image the vascular plexuses of the retina in great detail [8]. Jia et al. were the first to use OCTA for ONH blood flow and showed compromised macular and ONH VD in glaucoma patients [9]. Other studies have also shown evidence of VD loss in NTG and POAG patients [10,11,12].

In the present study, we aimed to compare the VD in various vascular beds in the macula using OCTA on patients suffering from NTG and POAG, along with healthy age matched participants. We also tested for the associations between OCTA VD parameters and OCT thickness parameters, as well as visual field indices.

## 2. Materials and Methods

This is a cross-sectional study with prospective recruitment consisting of three groups: POAG patients, NTG patients and healthy controls. The study protocol was approved by the Review Boards of the University Hospital of Larissa and the Papageorgiou Hospital of Thessaloniki, Greece. All subjects provided written informed consent and the principles of the Declaration of Helsinki were followed.

To be included in the study, glaucoma patients had to be suffering from glaucomatous optic neuropathy in the absence of signs of secondary glaucoma (i.e., pigmentary dispersion syndrome, pseudoexfoliation etc.) and repeatable visual field defects compatible with glaucoma. Glaucomatous optic neuropathy was defined as neuroretinal rim thinning or notching, RNFL loss or asymmetry >0.2 in cup-to-disc (C/D) ratio unexplained by differences in optic disc size, as evaluated by a glaucoma specialist. In the POAG group, a history of untreated IOP > 21 mmHg was required, while all NTG patients had documentation of an 24 h IOP curve with no event of untreated IOP higher than 21 mmHg. Inclusion criteria for healthy controls were absence of fundoscopical evidence of glaucomatous optic neuropathy, IOP < 21 mmHg and normal macular and ONH OCT. A gonioscopically open anterior chamber angle was also required in all subjects. Exclusion criteria for all subjects were systemic cardiovascular disease (apart from controlled systemic hypertension), history of intraocular surgery or trauma and any other concurrent ocular or systemic condition that could affect the macula or the optic nerve. All participants (in the glaucoma groups and the control group) were required to have a refraction of less than 3 diopters of myopia, less than 3 diopters of hyperopia and less than 2 diopters of astigmatism. Healthy controls with a history of ocular hypertension or family history of glaucoma were also excluded. All glaucoma patients were already receiving antiglaucoma medications.

Only one eye was analyzed for each patient. For glaucoma patients, the worse eye was selected, based on VF mean deviation (MD) score, while for healthy controls the study eye was randomly selected using random number generation via Microsoft Excel Spreadsheet Software (version 16.41, Microsoft Corporation, Redmond, WA, USA).

All subjects underwent full ophthalmic evaluation including slit-lamp biomicroscopy and fundoscopy, IOP measurement with Goldmann applanation tonometry, macular and ONH OCT and OCTA (Avanti XR with AngioVue module; Optovue Inc. Fremont, CA, USA). For macular imaging, the OCT GCC scan and HD Angio Retina [6.0] OCTA scan were used. For ONH imaging, we used the ONH OCT scan and the HD Angio Disc [4.5] OCTA scan. The parameters measured were ganglion cell complex (GCC) thickness, retinal nerve fiber layer (RNFL) thickness, SVP VD, DVP VD and CC VD. For calculation of CC VD, a slab between 10 and 30 microns below the retinal pigment epithelium was chosen in the HD Angio Retina scan. For each one of these parameters, we recorded the values for the inferior subfield and superior subfield, as well as the average value, based on the automated results of the AngioVue software (version 2018.1.0.43). Furthermore, we measured central corneal thickness and recorded the mean deviation (MD) values of the latest visual field test (Humphrey Field Analyzer, Carl Zeiss AG, Oberkochen, Germany) in all glaucoma patients.

OCT and OCTA were required to have a signal strength index over 60 and exhibit no gross artifacts. The acquisition was repeated in the case of a suboptimal scan and was rejected in case of inability to achieve an acceptable scan quality.

The distribution for quantitative variables was checked for normality with the Kolmogorov–Smirnov test. The mean ± standard deviation was calculated in the case of a normal distribution. The median (interquartile range) was recorded in case of skewed distributions. Comparisons were performed with the one-way ANOVA test. Bonferroni post hoc tests followed, in case of a significant *p*-value. Comparisons of measurements only between the two glaucoma groups were performed with the independent samples *t*-test. The sex distribution between the groups was tested with the chi square test. Correlations between metrics were tested with Pearson test. *p*-value was set at 0.05. Data were analyzed using SPSS software version 24 (SPSS Inc., Chicago, IL, USA).

## 3. Results

Thirty-three NTG, 65 POAG and 40 eyes in the control group were analyzed. The mean age was 72.5 ± 8.3 years in the NTG, 70.3 ± 7.4 years in the POAG and 68.7 ± 6.0 years in the control group (*p* = 0.084). There was no difference in sex distribution in the three groups (*p* = 0.482). All participants were Caucasian. Baseline characteristics of the groups are presented in Table 1.

All study images were inspected for quality and segmentation. No manual corrections were deemed necessary regarding the automatic layer segmentation. OCT NFL and GCC measurements were all significantly lower in the NTG and POAG groups compared to the control group. However, looking at the pairwise comparisons between the NTG and POAG, there was no difference in average, inferior or superior NFL and in average, inferior or superior GCC (see Table 2).

**Figure 1:**
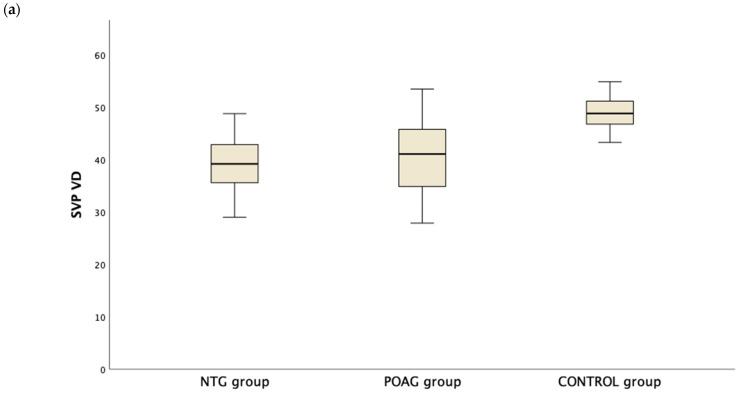

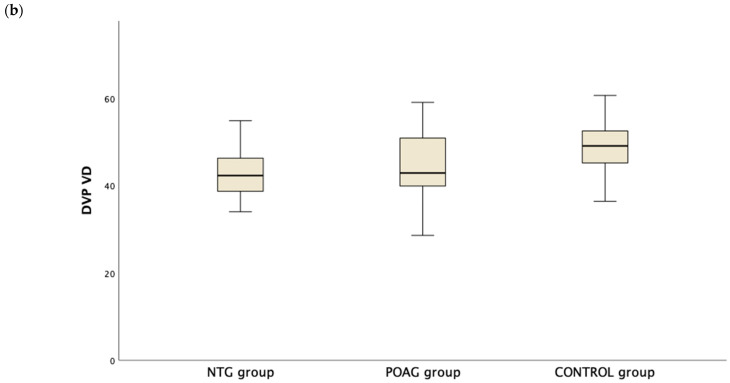
Boxplot of the vessel density (VD) in the superficial vascular plexus (SVP, (**a**)) and the deep vascular plexus (DVP, (**b**)) in the normal tension glaucoma (NTG), primary open angle glaucoma (POAG) and the control group.

**Figure 2:**
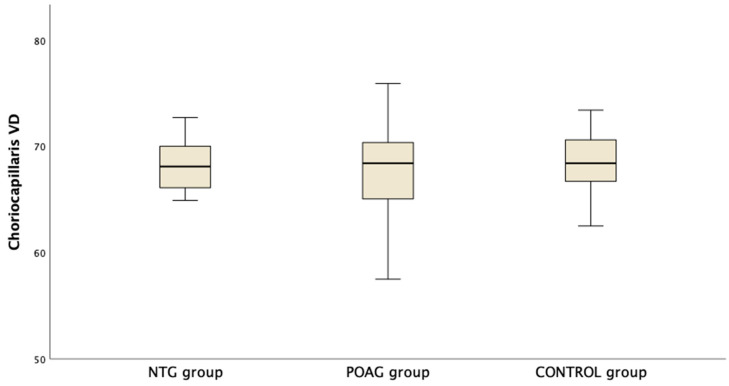
Boxplot of the vessel density (VD) in the choriocapillaris (CC) slab in the macula area in normal tension glaucoma (NTG), primary open angle glaucoma (POAG) and the control group.

Table 3 presents the OCTA measurements from the retina vascular plexi and the choriocapillaris layer. VD measurements were lower in the SVP and DVP (average, inferior and superior hemifield) VD when either one of the glaucoma groups was compared to the control group (Figure 1). Interestingly there was no difference in CC VD in any of the three parameters (including average, inferior and superior hemifield CC VD) (Figure 2).

Correlations were then tested between the OCTA parameters, OCT metrics and visual field indices. First, a significant correlation exists between OCTA SVP VD and either GCC or NFL in the OCT metrics (see Table 4) in all three groups. Furthermore, eyes with POAG showed a significant positive correlation between DVP VD and GCC/NFL, as well as MD. On the contrary, analysis failed to reveal such a correlation in eyes with NTG. In the control group, OCT thickness parameters were significantly correlated with SVP VD, not DVP VD or CC VD. CC VD was not related to any OCT or visual field parameter, SVP VD or DVP VD. SVP VD and DVP VD were positively correlated in all three groups.

## 4. Discussion

In this cross-sectional study, we prospectively recruited patients with POAG and NTG, along with healthy controls, and investigated the differences in macular VD between these groups. Our results show similar values between NTG and POAG patients and decreased VD in glaucoma patients compared to controls in both retinal slabs, superficial and deep, while no difference was detected in the CC slab measurements. Similar results were seen when comparing average, superior or inferior VD measurements. These differences highlight the vascular changes that occur with POAG and NTG compared to the healthy eye. However, the absence of clear differences in OCTA VD parameters between POAG and NTG matched for age and stage of glaucoma suggests that the two groups share similarities in terms of OCTA imaging. Therefore, there is no evidence from the OCTA imaging to support a more severely impaired vascular perfusion in eyes with NTG.

Interestingly, SVP VD is the parameter that characterizes the density of the perfused capillaries that nourish the innermost part of the retina. Other studies in the literature have also quantified SVP VD. Mursch Edlmayer et al. explored the superficial perfusion density in the central 3 mm of the macula with swept source OCTA and found no difference between NTG and high tension glaucoma [13]. Xu et al. found that total retina perfused VD was lower in both high tension glaucoma and NTG eyes [12]. VD in that study was measured for the whole retina, from the internal limiting membrane to the retinal pigment epithelium. In our study, both the SVP and DVP were measured in an area of 6 × 6 mm^2^. In the study by Lommatz et al., superficial and deep VD was compared between POAG, NTG and pseudoexfoliative glaucoma patients and no difference was detected [14]. Finally, based on their findings, Shen et al. proposed that the macular intercapillary area calculated from the OCTA images using custom software may be an important biomarker for glaucoma [15]. The authors reported that NTG patients had lower global VD compared to patients with healthy eyes and also that enlargement of the macular intercapillary area was associated with early central VF loss. Other parameters, like branching complexity and foveal avascular zone area, seem to be less useful [16].

It is true that most studies have focused on SVP measurements in glaucoma as opposed to DVP measurements. The latter characterize the deeper capillary network that is located below the inner nuclear layer and is supplied by vertical anastomoses from the SVP [17]. It is noteworthy that earlier studies were unable to differentiate SVP and DVP VD. However, it has recently become clear that DVP VD may also carry important information. The advancement of OCTA algorithms facilitated the accurate quantification of the retinal microvasculature, with more reliable results especially for the deeper layers [18]. Moreover, the association of decreased DVP with a higher risk of developing perimetric loss was recently reported and supports the role of DVP as an early biomarker of visual field loss [19]. Moreover, low DVP VD emerged as an independent risk factor for the development of a central scotoma, on top of structural OCT thinning in NTG patients [20].

SVP VD was the parameter from OCTA that correlated to both OCT NFL and GCC, as well as VF MD. Therefore, there was a close relationship between perfusion in the superficial slab and OCT structural thickness parameters as shown on the OCT scan, both in the macula and also in the ONH, as well as in terms of functional perimetric loss, when measured with MD. Patients with a more advanced stage of the disease also showed profound capillary loss. Interestingly, Kim et al. found that the topographic location of inner retina thinning co-localized with the location of vessel density loss on the OCTA in NTG [21]. They examined glaucoma suspect and early stage NTG eyes and found that sectoral macular VD measurements showed significant topographic correlations with respective macular thickness in the ganglion cell inner plexiform layer, as well as the relevant circumpapillary NFL thickness. SVP VD correlated with inner retina thickness in the study by Jeon et al., but DVP VD failed to show any association with OCT in NTG patients with a parafoveal scotoma [20]. In that study, DVP VD showed a significant correlation with MD in the SITA 10-2 VF exam. This is in line with the findings of our study, where DVP VD failed to show an association with OCT thickness parameters. We did not test patients with the 10-2 VF, but our findings did not support a correlation between DVP VD and 24-2 MD. However, in our study, we did find a correlation between DVP VD and GCC, NFL and MD in the POAG group. Therefore, an association between VD loss, GCC thinning and decrease in MD exists, but the cross-sectional design of the study cannot provide further insight to the chronological series of events. Onishi et al. reported that the decrease in both macular and peripapillary VD predated significant GCC and RNFL damage in early glaucoma [22]. In fact, structural thinning and a decrease in VD has been detected in perimetrically normal hemifields of eyes with NTG [23]. It is now believed that OCTA may complement OCT and VF.

In this study we also examined for possible differences in the choriocapillaris slab in patients with NTG and POAG and in healthy subjects. Interestingly, studies on the macula choroid in glaucoma are limited. Choroidal blood flow has been indirectly measured and parameters such as choroidal thickness and choroidal vascularity index have been used as proxies for blood flow. Choroidal thickness represents the thickness of the whole choroid and not only the innermost layer of choriocapillaris, as is the case in our study that measured VD in a small slab that hosts the choriocapillaris layer. Wang et al. used OCT to characterize the choroid in the macula in a large cohort of NTG patients and controls [24]. They analyzed the thickness and choroidal vascularity index (CVI) and found that CVI in the Haller’s layer, which is the part of the choroid that accommodates the larger vessels, is reduced compared to healthy eyes and correlated with the degree of glaucoma damage. Therefore, they concluded that Haller’s layer, more than the choriocapillaris or Sattler’s layer, is affected in NTG. In another study, the authors reported a lower CVI in POAG patients compared to controls [25].

Only a few studies have reported on CC VD in glaucoma with OCTA imaging. Tepelus et al. used swept-source (SS) OCTA to image eyes with NTG and healthy controls and found that eyes with NTG had a lower CC perfusion density, as well as a lower SVP and peripapillary vessel length density compared to normal eyes [26]. Cennamo et al. also compared eyes with preperimetric glaucoma, advanced glaucoma and controls [27]. However, the type of open angle glaucoma in those patients was not reported. They used enhanced depth imaging—OCT and OCTA—and found that CC VD was reduced in glaucoma patients compared to controls. In our study, the choriocapillaris layer was studied, the groups were clearly defined and spectral domain OCTA was utilized, and we did not find any difference between the groups. In addition, CC VD in our study showed no association to OCT structural parameters or perimetric MD or to SVP or DVP MD. Recently, Cheng et al. reported their findings from a large study in non-glaucomatous eyes and found a negative correlation between CC flow deficit percentage (FD%) with SS OCTA and GCIPL, meaning that a less dense choroidal microcirculation is associated with thinner inner retinal layer thickness [28]. This was supported by both the cross-sectional and the longitudinal study that showed that a faster increase in CC FD% was found to be significantly associated with a higher risk of neurodegeneration of the inner retina. Therefore, more studies are needed to better characterize the information that CC VD carries and the association of CC VD with various types of glaucoma.

OCT NFL and GCC thickness metrics uniformly measured lower in glaucoma patients compared to the control group. This is expected since GCC and NFL damage are linked to glaucomatous optic neuropathy and apoptosis of the neural tissue [29]. They were, however, similar in the two glaucoma groups in the present study. This is especially important since the staging of glaucoma is based on VF and these structural parameters can change with disease progression at a different rate compared to the visual field, while their change may be detected at a different time compared to perimetry [30]. It is, therefore, important that in the present study the two glaucoma groups had similar OCT measurements, on top of the similar values for MD, rendering the two groups alike when comparing as to their microvascular properties.

Certain limitations should be acknowledged when looking at the results of the present study. The relatively small sample and variable antiglaucoma medication comprise some of the weaknesses of this study. All glaucoma patients were treated with topical therapy at the time of measurements and had IOPs within the normal range. However, this is typical for similar studies in the literature. Nonetheless, this is one of the few studies that excluded secondary open angle glaucoma while assessing the participation of multiple vascular plexi in glaucoma. Indeed, the study quantified both the SVP and DVP in the retinal vasculature and also the CC VD, which characterizes the innermost part of the choroid. Moreover, one of the strengths of this study relates to the strict criteria used for the diagnosis of NTG, which was based on a twenty-four-hour profiling of the IOP. Furthermore, the stage of glaucoma damage was similar in NTG and POAG patients. Imaging was performed in an area of 6 × 6 mm, which is thought to capture more peripheral changes compared to the 3 × 3 mm area that was used in some of the studies in the literature. In addition, only one eye per patient was included, as per the protocol of the study. Only Caucasian patients were recruited.

## 5. Conclusions

In conclusion, OCT angiography vessel density measurements of the retina in the macula are lower in eyes with primary open angle or normal tension glaucoma compared to control subjects. Vessel density in the choriocapillaris layer does not differ between glaucoma patients and controls. However, vessel density in the superficial and the deep vascular plexus of the retina is similar in (high pressure) primary open angle and normal tension glaucoma of a similar stage in eyes that are treated with glaucoma drops. Since, the temporal relationship of vascular perfusion changes and retinal structural thinning of the ganglion cells and their axons remains largely unclear up to this date, further research in this direction may help clarify the chronological series of events in each glaucoma subtype.

## Figures and Tables

**Table 1:** Baseline characteristics of the normal tension glaucoma (NTG) group, primary open angle glaucoma (POAG) group and the control group. *p* values are presented for the overall one-way ANOVA, followed by pairwise comparisons with the Bonferroni correction.

	NTG Group (*n* = 33)	POAG Group (*n* = 65)	Control Group (*n* = 40)	*p*-Value
Age	72.5 ± 8.3	70.3 ± 7.4	68.7 ± 6.0	Overall: 0.084 (1)–(2): 0.461 (1)–(3): 0.811 (2)–(3): 0.080
Sex (men/women)	17/16	26/39	16/24	Overall: 0.482 ^1^
MD (dB)	−8.71 ± 7.11	−6.47 ± 6.81		(1)–(2): 0.192 ^2^
PSD (dB)	6.92 ± 4.52	4.93 ± 3.69		(1)–(2): 0.047 ^2^
C/d ratio	0.83 ± 0.11	0.75 ± 0.18	0.37 ± 0.12	Overall: <0.001 (1)–(2): 0.064 (1)–(3): <0.001 (2)–(3): <0.001
CCT	522.9 ± 36.7	539.5 ± 40.7		(1)–(2): 0.061 ^2^

^1^ chi square test. ^2^ independent samples *t*-test. MD: mean deviation, PSD: pattern standard deviation, C/d: cup-to-disc and CCT: central corneal thickness.

**Table 2:** OCT measurements in the normal tension glaucoma (NTG) group, primary open angle glaucoma (POAG) group and the control group. *p* values are presented for the overall one-way ANOVA, followed by pairwise comparisons with the Bonferroni correction.

		NTG Group (*n* = 33)	POAG Group (*n* = 65)	Control Group (*n* = 40)	*p*-Value
RNFL	Average	76.8 ± 14.0	79.5 ± 14.1	97.9 ± 7.9	Overall: <0.001 (1)–(2): 0.942 (1)–(3): <0.001 (2)–(3): <0.001
	Inferior	76.1 ± 15.5	77.6 ± 14.7	95.5 ± 8.2	Overall: <0.001 (1)–(2): 0.595 (1)–(3): <0.001 (2)–(3): <0.001
	Superior	77.6 ± 14.7	81.4 ± 14.9	100.5 ± 9.9	Overall: <0.001 (1)–(2): 0.579 (1)–(3): <0.001 (2)–(3): <0.001
GCC	Average	79.2 ± 15.0	81.5 ± 15.1	95.1 ± 8.2	Overall: <0.001 (1)–(2): 1.0 (1)–(3): <0.001 (2)–(3): <0.001
	Inferior	80.6 ± 14.9	80.2 ± 15.7	95.6 ± 7.9	Overall: <0.001 (1)–(2): 1.0 (1)–(3): <0.001 (2)–(3): <0.001
	Superior	77.7 ± 16.8	82.7 ± 16.4	94.7 ± 9.2	Overall: <0.001 (1)–(2): 0.365 (1)–(3): <0.001 (2)–(3): <0.001

**Table 3:** OCTA measurements at the superficial vascular plexus (SVP), the deep vascular plexus (DVP) and the choriocapillaris (CC) slab in the normal tension glaucoma (NTG) group, primary open angle glaucoma (POAG) group and the control group. *p* values are presented for the overall one-way ANOVA, followed by pairwise comparisons with the Bonferroni correction.

		NTG Group (*n* = 33)	POAG Group (*n* = 65)	Control Group (*n* = 40)	*p*-Value
SVP VD	Average	38.8 ± 5.3	40.7 ± 6.8	48.5 ± 4.0	Overall: <0.001 (1)–(2): 0.380 (1)–(3): <0.001 (2)–(3): <0.001
	Inferior	38.6 ±5.7	40.0 ± 7.3	48.5 ± 3.9	Overall: <0.001 (1)–(2): 0.162 (1)–(3): <0.001 (2)–(3): <0.001
	Superior	38.9 ± 5.4	41.2 ± 6.5	48.5 ± 4.2	Overall: <0.001 (1)–(2): 0.848 (1)–(3): <0.001 (2)–(3): <0.001
DVP VD	Average	43.1 ± 6.1	44.5 ± 7.6	48.6 ± 5.8	Overall: 0.002 (1)–(2): 0.960 (1)–(3): <0.001 (2)–(3): 0.001
	Inferior	42.9 ± 6.5	43.8 ± 8.0	48.6 ± 6.1	Overall: <0.001 (1)–(2): 1.000 (1)–(3): <0.001 (2)–(3): <0.001
	Superior	43.2 ± 6.2	45.2 ± 7.5	48.5 ± 6.1	Overall: 0.004 (1)–(2): 0.497 (1)–(3): 0.003 (2)–(3): 0.049
CC VD	Average	68.3 ± 2.3	67.6 ± 3.7	68.5 ± 2.6	Overall: 0.287
	Inferior	68.1 ± 2.6	67.6 ± 3.9	68.3 ± 2.9	Overall: 0.119
	Superior	68.5 ± 2.4	67.6 ± 3.7	68.8 ± 2.7	Overall: 0.593

**Table 4:** Results of univariate regression analysis showing Pearson r (*p*-value) between parameters from OCTA, OCT and visual fields. Results are reported for the NTG (normal tension glaucoma), POAG (primary open angle glaucoma) and control group.

	GCC	NFL	MD	DVP VD	CC VD
NTG group					
SVP VD	0.551 (0.001)	0.744 (<0.001)	0.395 (0.042)	0.580 (<0.001)	
DVP VD	0.215 (0.245)	0.167 (0.369)	0.118 (0.592)		
CC VD	−0.169 (0.372)	−0.414 (0.019)	−0.225 (0.290)		
POAG group					
SVP VD	0.621 (<0.001)	0.741 (<0.001)	0.656 (<0.001)	0.733 (<0.001)	0.004 (0.974)
DVP VD	0.335 (0.007)	0.347 (0.006)	0.304 (0.038)		0.046 (0.726)
CC VD	−0.211 (0.102)	−0.125 (0.342)	0.035 (0.820)		
CONTROL group					
SVP VD	0.511 (<0.001)	0.429 (0.006)		0.646 (<0.001)	−0.047 (0.775)
DVP VD	0.234 (0.145)	0.272 (0.090)			−0.211 (0.192)
CC VD	0.294 (0.065)	0.346 (0.249)			

VD: vessel density, SVP: superficial vascular plexus, DVP: deep vascular plexus, CC: choriocapillaris, GCC: ganglion cell complex, NFL: nerve fiber layer and MD: mean deviation.

## Data Availability

Data are available on reasonable request.

## References

[1] Bourne R.R.A., Jonas J.B., Friedman D., Nangia V., Bron A., Tapply I., Fernandes A., Cicinelli M.V., Arrigo A., Vision Loss Expert Group of the Global Burden of Disease Study (2024). Global estimates on the number of people blind or visually impaired by glaucoma: A meta-analysis from 2000 to 2020. Eye.

[2] Cho H., Kee C. (2014). Population-based glaucoma prevalence studies in Asians. Surv. Ophthalmol..

[3] Gasser P., Flammer J. (1991). Blood-Cell Velocity in the Nailfold Capillaries of Patients with Normal-Tension and High-Tension Glaucoma. Am. J. Ophthalmol..

[4] Plange N., Kaup M., Huber K., Remky A., Arend O. (2006). Fluorescein filling defects of the optic nerve head in normal tension glaucoma, primary open-angle glaucoma, ocular hypertension and healthy controls. Ophthalmic Physiol. Opt..

[5] Hamard P., Hamard H., Dufaux J., Quesnot S. (1994). Optic nerve head blood flow using a laser Doppler velocimeter and haemorheology in primary open angle glaucoma and normal pressure glaucoma. Br. J. Ophthalmol..

[6] Stalmans I., Harris A., Fieuws S., Zeyen T., Vanbellinghen V., McCranor L., Siesky B. (2009). Color Doppler Imaging and Ocular Pulse Amplitude in Glaucomatous and Healthy Eyes. Eur. J. Ophthalmol..

[7] Zhang A., Zhang Q., Chen C.-L., Wang R.K. (2015). Methods and algorithms for optical coherence tomography-based angiography: A review and comparison. J. Biomed. Opt..

[8] Campbell J.P., Zhang M., Hwang T.S., Bailey S.T., Wilson D.J., Jia Y., Huang D. (2017). Detailed Vascular Anatomy of the Human Retina by Projection-Resolved Optical Coherence Tomography Angiography. Sci. Rep..

[9] Jia Y., Morrison J.C., Tokayer J., Tan O., Lombardi L., Baumann B., Lu C.D., Choi W., Fujimoto J.G., Huang D. (2012). Quantitative OCT angiography of optic nerve head blood flow. Biomed. Opt. Express.

[10] Bojikian K.D., Chen C.-L., Wen J.C., Zhang Q., Xin C., Gupta D., Mudumbai R.C., Johnstone M.A., Wang R.K., Chen P.P. (2016). Optic Disc Perfusion in Primary Open Angle and Normal Tension Glaucoma Eyes Using Optical Coherence Tomography-Based Microangiography. PLoS ONE.

[11] Arish M., Momeni-Moghaddam H., Alborzi M., Maleki A., Daneshvar R., Heidari H.-R. (2023). Peripapillary vessel density in healthy people, primary open-angle glaucoma, and normal-tension glaucoma. Eur. J. Ophthalmol..

[12] Xu H., Zhai R., Zong Y., Kong X., Jiang C., Sun X., He Y., Li X. (2018). Comparison of retinal microvascular changes in eyes with high-tension glaucoma or normal-tension glaucoma: A quantitative optic coherence tomography angiographic study. Graefe’s Arch. Clin. Exp. Ophthalmol..

[13] Mursch-Edlmayr A.S., Waser K., Podkowinski D., Bolz M. (2020). Differences in swept-source OCT angiography of the macular capillary network in high tension and normal tension glaucoma. Curr. Eye Res..

[14] Lommatzsch C., Rothaus K., Koch J., Heinz C., Grisanti S. (2019). Vessel Density in Glaucoma of Different Entities as Measured with Optical Coherence Tomography Angiography. Clin. Ophthalmol..

[15] Shen R., Wang Y.M., Cheung C.Y., Tang F.Y., Lam A., Tham C.C., Chan P.P. (2022). Relationship between macular intercapillary area measured by optical coherence tomography angiography and central visual field sensitivity in normal tension glaucoma. Br. J. Ophthalmol..

[16] CCheng K.K.W., Tan B.L., Brown L., Gray C., Bianchi E., Dhillon B., MacGillivray T., Tatham A.J. (2021). Macular vessel density, branching complexity and foveal avascular zone size in normal tension glaucoma. Sci. Rep..

[17] Provis J.M. (2001). Development of the Primate Retinal Vasculature. Prog. Retin. Eye Res..

[18] Takusagawa H.L., Liu L., Ma K.N., Jia Y., Gao S.S., Zhang M., Edmunds B., Parikh M., Tehrani S., Morrison J.C. (2017). Projection-Resolved Optical Coherence Tomography Angiography of Macular Retinal Circulation in Glaucoma. Ophthalmology.

[19] Lin S., Shang X., Wang X., Chu X., Hu C., Si Y., Chen D.-F., Zhou W., Kong Y.X.G., Liang Y. (2022). Decreased macular deep capillary plexus is associated with functional progression of normal tension glaucoma patients with unilateral visual field loss. Br. J. Ophthalmol..

[20] Jeon S.J., Park H.-Y.L., Park C.K. (2018). Effect of Macular Vascular Density on Central Visual Function and Macular Structure in Glaucoma Patients. Sci. Rep..

[21] Kim J.-S., Kim Y.K., Baek S.U., Ha A., Kim Y.W., Jeoung J.W., Park K.H. (2019). Topographic correlation between macular superficial microvessel density and ganglion cell-inner plexiform layer thickness in glaucoma-suspect and early normal-tension glaucoma. Br. J. Ophthalmol..

[22] Onishi A.C., Treister A.D., Nesper P.L., A Fawzi A., Anchala A.R. (2019). Parafoveal vessel changes in primary open-angle glaucoma and normal-tension glaucoma using optical coherence tomography angiography. Clin. Ophthalmol..

[23] Uchida N., Ishida K., Anraku A., Takeyama A., Tomita G. (2019). Macular vessel density in untreated normal tension glaucoma with a hemifield defect. Jpn. J. Ophthalmol..

[24] Wang Y.M., Hui V.W.K., Shi J., Wong M.O.M., Chan P.P., Chan N., Lai I., Cheung C.Y., Tham C.C. (2021). Characterization of macular choroid in normal-tension glaucoma: A swept-source optical coherence tomography study. Acta Ophthalmol..

[25] Wang D., Xiao H., Lin S., Fang L., Gan Y., Zhang Y., Chen X., Huang Z., Zheng S., Shi H. (2023). Comparison of the Choroid in Primary Open Angle and Angle Closure Glaucoma Using Optical Coherence Tomography. J. Glaucoma.

[26] Tepelus T.C., Song S., Borrelli E., Nittala M.G., Baghdasaryan E., Sadda S.R., Chopra V. (2019). Quantitative Analysis of Retinal and Choroidal Vascular Parameters in Patients With Low Tension Glaucoma. J. Glaucoma.

[27] Cennamo G., Malvone E., Marotta M., Breve M.A., Costagliola C. (2022). Study of choroidal vasculature in open angle glaucoma patients. Photodiagnosis Photodyn. Ther..

[28] Cheng W., Wang W., Song Y., Lin F., Duan Y., Xie L., Jin K., Weinreb R.N., Zhang X. (2022). Choriocapillaris and progressive ganglion cell-inner plexiform layer loss in non-glaucomatous eyes. Br. J. Ophthalmol..

[29] Bussel I.I., Wollstein G., Schuman J.S. (2014). OCT for glaucoma diagnosis, screening and detection of glaucoma progression. Br. J. Ophthalmol..

[30] Zhang X., Dastiridou A., Francis B.A., Tan O., Varma R., Greenfield D.S., Schuman J.S., Huang D. (2017). Comparison of Glaucoma Progression Detection by Optical Coherence Tomography and Visual Field. Am. J. Ophthalmol..

